# Addressing Common Misuses and Pitfalls of *P* values in Biomedical Research

**DOI:** 10.1158/0008-5472.CAN-21-2978

**Published:** 2022-08-03

**Authors:** Ming Wang, Qi Long

**Affiliations:** 1 Division of Biostatistics and Bioinformatics and Penn State Cancer Institute, Penn State College of Medicine, Hershey, Pennsylvania.; 2 Department of Biostatistics, Epidemiology and Informatics and Abramson Cancer Center, University of Pennsylvania, Philadelphia, Pennsylvania.

## Abstract

In recent years, there has been a growing recognition that *P* values, albeit useful in reporting data analysis results, have often been misused or misinterpreted in biomedical research. The emergence of big health data such as genomics data and electronic health records, sometimes combined with inadequate experimental design, has exacerbated this problem, which has become a major cause of the ongoing crisis in reproducibility in biomedical research. We aim to shed light and raise awareness of common misuses and pitfalls of *P* values and discuss potential mitigation strategies that leverage state-of-the-art statistical methods. The best practices always start with a sound study design including a robust data collection strategy to minimize data bias and a carefully thought-out analysis plan that can address potential misuses and pitfalls of *P* values. We highly encourage biomedical researchers to engage and involve statisticians from the very beginning of their studies.

## Introduction

*P* values, commonly used for quantifying evidence against a null hypothesis in statistical inference, have served an important role in biomedical research. Originated since the 1700s, *P* values became popularized in the 1920s, and later, their utilizations have been gradually morphed and deviated from original definitions. *P* value measuring statistical significance is defined as “the probability under a specified statistical model that a statistical summary of the data would be equal to or more extreme than its observed value” (1). Thus, given the null and alternative hypotheses as well as the observe data, *P* value can be calculated on the basis of specific test statistics to evaluate the evidence rejecting the null hypothesis. Despite there exists a clear definition with rigorous statistical properties, *P* values have been misused or misinterpreted along with hypothesis testing by researchers due to multifarious reasons. In recent years, there have been vigorous debates about *P* values including its limitations and flaws. The American Statistical Association (ASA) has put out statements on *P* values with warnings against its misuses and suggestions on alternatives and complements to *P* values (1, 2). In parallel, leading scientific journals (e.g., *New England Journal of Medicine*) and government agencies (e.g., the United States Food and Drug Administration) have also attempted to provide specific guidelines on reporting *P* values (3). Despite its limitations and flaws, many have argued that *P* values can and should still play a valuable role in statistical analysis, which can be complemented by other alternative approaches. As such, it remains critical to recognize common misuses of *P* values in biomedical research and understand appropriate mitigation strategies. To this end, we highlight several notable examples largely based on our experience in cancer research (Table 1).

**Table 1.** Key concepts, examples, and potential solutions for misuses and pitfalls of *P* values.

Misuses and pitfalls of *P* values	Examples in cancer research	Potential solutions
Overemphasizing statistical significance over clinical significance	Small *P* value and small effect size estimate in analysis of a large cancer data set from cancer registry or EHR	Report the estimate of effect size and associated confidence interval; consider whether the estimated effect size is clinically significant
Mis-specified models or inappropriate testing procedures	Conducting parametric tests in analysis of animal studies in cancer research with small sample size	Assess and confirm data normality first before applying normality-based methods; or apply more robust methods such as nonparametric tests
Inflated Type I error due to multiple comparisons	Assessing association between gene expression levels and cancer patient survival outcome using data from TCGA	Use methods for controlling FDR or familywise error rate etc. as appropriate
Misinterpretation of *P* > 0.05	Concluding that two cancer treatments are equally effective when *P* > 0.05 for testing a null hypothesis that the outcomes in two treatment groups are the same	Consider equivalence or non-inferiority/superiority tests
*P* value in analysis of observational data	Significant *P* value in analysis of cancer registry data, where confounding is not adequately accounted for	Use appropriate methods for adjusting for confounding and other sources of bias

### Clinical/scientific significance (effect size) versus statistical significance

Statistical significance is not equivalent to clinical or scientific significance. The original intention of *P* values is to present the extent of evidence on whether an association study needs further scrutiny or not. The magnitude of *P* values does not reflect the size of an association or effect. An assertion with simple dichotomization of “significant” and “not significant” based on some threshold (e.g., 0.05) for *P* values without any other supplemental information (e.g., confidence intervals, effect size) is somewhat arbitrary and may not be scientifically informative or meaningful. In addition, large changes in *P* values can correspond to small, nonsignificant changes in the underlying quantities such as treatment effect estimates, suggesting a clear distinction between scientific significance and statistical significance.

Many factors may impact the evidence for rejecting a null hypothesis including, but not limited to, study design, sampling scheme, data collection process, and analytical models. It is particularly notable that *P* value is a function of sample size (*N*), with larger *N* leading to smaller *P* values. Thus, the boundary of significance level can be eventually achieved against the null hypothesis, as *N* becomes sufficiently large, which has become increasingly common in the era of big data. For instance, national cancer registry data (e.g., the National Cancer Database; the Surveillance the Epidemiology, and End Results Database) or electronic health records (EHR) data have been widely used in cancer research for say, uncovering patient heterogeneity and thus identifying potential risk factors with the ultimate goal of improving cancer outcomes. In such analyses, *P* values could be very small. However, if the effect size (i.e., the point estimate of association or treatment effect) is close to the null value, then the findings may not be clinically meaningful or significant despite small *P* values (Table 1) and such findings are also less likely to be reproducible due to potential biased estimation. As the sample size increases, model misspecification and estimation biases become much more significant concerns; in other words, if the effect size estimate is biased, then its associated *P* value is not meaningful. To address these concerns, it is recommended that confidence intervals should be presented along with point estimates of associations or effects along with their *P* values to support main conclusions.

### Use of mis-specified models or inappropriate testing procedures

*P* values are typically computed for a specific model or statistical testing procedure based on its theoretical properties. Thus, if the model or testing procedure used is mis-specified or the assumptions underlying the methods are violated for the data being analyzed, the resulting *P* values would not be valid and would lead to flawed conclusions. This issue in our experience has not received sufficient attention in practice. For example, this issue is particularly relevant in animal studies in cancer research with small to moderate sample size in each individual experiment (Table 1). To analyze such data, one needs to give careful considerations about appropriateness of statistical models and hypothesis testing procedures. Some statistical models and testing procedures such as linear regression, ANOVA, and two-sample *t* tests are valid under the assumption that the data are normally distributed and/or based on asymptotic results that require a large sample size to be valid. If these conditions and assumptions do not hold (e.g., the data are not normally distributed or have extreme values), then the resulting *P* values are invalid. Instead, nonparametric tests should be considered (e.g., Wilcoxon rank-sum tests for two-group comparison on continuous or ordinal variables; Kruskal–Wallis tests for more than two groups’ comparison on continuous or ordinal variables; Fisher exact tests for categorical variables). In addition, parametric regression models are sensitive to mis-specification. Alternatively, semi-parametric or nonparametric regression models can be used to obtain more robust results. For instance, generalized estimating equations is a popular semi-parametric approach to make marginal inference on longitudinal data, yielding more robust results than classical mixed-effect models. Also, nonparametric approaches such as those based on empirical likelihood have drawn an increasing interest in data analysis. The robustness of the findings can be further assessed by performing *post hoc* and sensitivity analysis with a variety of analysis approaches.

### Inflated Type I error due to multiple comparisons

Another common misuse of *P* values is inflated Type I error (false positive) due to multiple comparisons/tests. As the number of simultaneous tests increases, it is well known that the probability of getting at least one statistically significant result due to random chances would also increase, leading to a higher level of false positives. Sometimes, this misuse can be subtle and not obvious. For example, many basic science cancer studies include multiple experiments for which statistical analyses are conducted for each experiment with the same threshold level for *P* values (say, *P* < 0.05) separately. In the past two decades, this misuse has become increasingly common and problematic as rapid advances in technologies have enabled collection of high-dimensional features in cancer research, e.g., tens of thousands of gene expression features or millions of SNP features in The Cancer Genome Atlas (TCGA). Oftentimes, univariate analysis is performed for each individual feature to identify important features associated with an outcome of interest, say cancer. Without proper adjustment for multiple comparisons, such analysis can lead to a substantially higher type I error than the prespecified significance level, e.g., α = 0.05 (Table 1), contributing to lack of reproducibility in biomedical research. To address this issue, it is important to conduct multiple testing correction on the *P* values for all tests to control the overall error rate [e.g., false discovery rate (FDR), familywise error rate] within a prespecified threshold. There exist many types of correction methods, including, Bonferroni, Bonferroni Step-down (Holm), Westfall and Young Permutation, Benjamini-Hochberg FDR, among others (4). These methods have different operating characteristics, thus the selection of appropriate multiple testing correction depends on specific data setting and analytical needs. For instance, the Bonferroni correction, known to be highly conservative in controlling false positives, tends to yield high false negatives and should be avoided when the number of tests is large. In comparison, the FDR approach tends to be less conservative and provides a good balance between limiting false negatives versus limiting false positives, particularly when the number of tests is large.

### What does *P* value > 0.05 mean?

In practice, the threshold level (α) for a statistical test is often set at 0.05. Statistical significance decision is made by comparing a *P* value with α. While most researchers understand the meaning of *P* value <0.05, many tend to misunderstand and misinterpret the meaning of *P* > 0.05 (1). Arguably the most common misinterpretation is interpreting *P* value >0.05 as that the null hypothesis is true and then concluding that say, there is no association between a risk factor and a disease such as cancer, or there is no treatment effect difference between two interventions that are compared in a cancer clinical trial (Table 1). Other common mistakes include interpreting *P* value as the probability that the null hypothesis is true. To help understand the meaning of *P* value >0.05, it is important to note that *P* value is calculated given that the null hypothesis is true, and does not rely on the alternative hypothesis. Thus, *P* value can be used to reject or fail to reject the null hypothesis, but cannot be used to accept the null hypothesis. In the case of nonsignificant *P* values, it is recommended to report the actual value of *P* value because the magnitude could still inform the precision and the extent of supporting evidence (2). Further, in order to show that there is no difference between two interventions in a clinical study, a valid approach is to conduct statistical tests for equivalence or non-inferiority/superiority, which tests whether the difference between the two treatment effects is less than a prespecified threshold that is considered clinically insignificant (5). Similarly, to formally show that there is no association between a biomarker and a clinical outcome, a valid approach is to test whether the magnitude of association is less than a prespecified threshold that is considered clinically insignificant (e.g., 0.02).

### Model selection and *P* values

Model selection plays a very important role in data analysis for biomedical research. *P* values are often used in practice as a criterion in model selection procedures such as forward, backward, and stepwise variable selection in regression analysis. However, *P* values are not designed for measuring the goodness of model fit and cannot provide any evidence in favor of a candidate model versus the other models. In addition, *P* values cannot be used for comparing models that are not nested within one another or selection of nuisance parameters. As such, it is not recommended to use *P* values in model selection. Instead, one can use well-established information criteria for model selection, such as Akaike information criterion (AIC), Bayesian information criterion (BIC), and Generalized Information Criteria (GIC), among others (6), which are typically designed to account for the trade-off between model fit and model complexity and hence mitigate overfitting in training data. Most of these criteria are likelihood-based information criteria, thus, if the distribution is mis-specified, their performance could deteriorate. To mitigate this issue, one could consider empirical likelihood (EL)–based information criteria such as empirical information criterion (EIC), empirical AIC and BIC, EL-based consistent information criterion, and others. Empirical likelihood, a nonparametric method, requires fewer assumptions about the error distribution than the classical likelihood approach; at the same time EL retains many appealing properties of likelihood-based inference. After model selection, one can fit the selected model and then apply standard techniques for computing *P* values for the final model.

In the case of regression models with high-dimensional features such as genomics data from the TCGA, it is considerably more challenging to perform model selection and compute *P* values for regression coefficients. One useful approach is penalized regression developed for high-dimensional data, e.g., least absolute shrinkage and selection operator (LASSO), elastic net, the smoothly clipped absolute deviation, adaptive LASSO, and group LASSO (7). These methods can achieve simultaneous model selection and estimation. However, it is not straightforward to obtain *P* values and confidence intervals for their regression coefficient estimates. There has been ongoing effort on addressing this challenge (8).

### *P* value in analysis of observational data

The aforementioned pitfalls of *P* values are especially acute in analysis of observational data e.g., data from cancer registries or EHR. Such analyses typically suffer from various types of bias, such as selection bias due to measured and unmeasured confounding, or immortal time bias. Sophisticated modeling approaches that often are needed to address such biases are subject to mis-specification. The resulting *P* values from mis-specified models, as a result of inappropriate handing of such biases, may not be meaningful (Table 1). In addition, unlike clinical trials and basic science experiments that are designed to test specific hypotheses with a prespecified analysis plan including correction for multiple comparisons, analyses of observational data in practice often involve an exploratory analysis step that leads to reporting cherry-picked strongest associations. This problem has been known as selective inference, “the assessment of significance and effect sizes from a dataset after mining the same data to find these associations” (9); to address this problem, one needs to set a higher threshold for declaring significant the associations estimated from the data. In addition, practitioners typically conduct many tests in analysis of observational data that are not preplanned, further worsening the issue of multiple comparisons. For these and other reasons, there have been reported contradiction or disagreement between randomized trials and observations studies in oncology (10), exacerbating the current crisis in reproducibility in biomedical research.

## Conclusion

We highlight several common misuses and pitfalls of *P* values and present potential mitigation strategies that can enhance reproducibility in biomedical research. The best practices start with a sound study design for all types of studies (i.e., prospective, retrospective, randomized and observational studies) including a carefully thought-out analysis plan. In the data analysis step, it is vital to identify appropriate statistical models and tests for the data being analyzed, properly address potential confounding and biases, and correct for multiple comparisons. Sensitivity analyses can be conducted to assess robustness of significant findings. When reporting analysis results, it is important to provide point estimates and confidence intervals (or other metrics of uncertainty quantification) in addition to associated *P* values, place more emphasis on clinical or scientific significance, and provide sufficient information for others to replicate the data analyses and reproduce the results. We highly encourage biomedical researchers to engage and involve statisticians from the very beginning of their studies to ensure a sound study design and a robust data collection and data analysis plan that can address misuses and pitfalls of *P* values including, but not limited to, those discussed in this article, and follow the guidelines on statistical analysis and reporting published by the ASA (1, 2) and *New England Journal of Medicine* (3).

### Data availability statement

Data sharing is not applicable to this article as no data were created or analyzed in this study.
